# Local resection versus radical resection after neoadjuvant chemoradiotherapy for patients with locally advanced rectal cancer: a propensity-score matched cohort analysis

**DOI:** 10.1186/s12876-023-02809-0

**Published:** 2023-06-13

**Authors:** Guancong Wang, Kaiyuan Yao, Yugang Yang, Hongying Chen, Zihan Tang, Jiahong Ye, Muhai Fu, Xiajuan Xue, Qiyuan Shen, Haiwen Tang, Yincong Guo, Ying Huang

**Affiliations:** 1 Department of Colorectal and Anal Surgery, Zhangzhou Affiliated Hospital of Fujian Medical University, Zhangzhou, 363000 China; 2 Department of Colorectal Surgery, Fujian Medical University Union Hospital, Fuzhou, 350001 China

**Keywords:** Locally advanced rectal cancer, Neoadjuvant chemoradiotherapy, Local resection, Propensity-score matched, Prognosis

## Abstract

**Background:**

We aimed to address the shortage of evidence regarding the safety of the local resection approach by comparing long-term oncological outcomes between patients managed by local resection and those who underwent radical resection.

**Methods:**

This was a propensity-score matched cohort analysis study that included patients of all ages diagnosed with locally advanced rectal cancer (LARC) who had received neoadjuvant chemoradiotherapy (nCRT) at the Fujian Medical University Union Hospital and Fujian Medical University Affiliated Zhangzhou Hospital, China, between Jan 10, 2011, to Dec 28, 2021. Partial patients with a significant downstage of the tumor were offered management with the local resection approach, and most of the rest were offered radical resection if eligible.

**Findings:**

One thousand six hundred ninety-three patients underwent radical resection after nCRT, and another 60 patients performed local resection. The median follow-up times were 44.0 months (interquartile range = 4–107 months). After propensity-core matching (PSM), in the Kaplan–Meier curves, local resection (*n* = 56) or radical resection (*n* = 211) was not significantly associated with 1-, 3-, and 5-year cumulative incidence of overall survival (OS) (HR = 1.103, 95% CI: 0.372 ~ 3.266), disease-free survival (DFS) ((HR = 0.972, 95% CI: 0.401 ~ 2.359), local recurrence (HR = 1.044, 95% CI: 0.225 ~ 4.847), and distant metastasis (HR = 0.818, 95% CI: 0.280 ~ 2.387) (all log-rank *P* > 0.05). Similarly, multivariate Cox regression analysis indicates that local excision still was not an independent risk factor for OS (HR = 0.863, 95% CI: 0.267 ~ 2.785, *P* = 0.805) and DFS (HR = 0.885, 95% CI: 0.353 ~ 2.215, *p* = 0.794).

**Conclusion:**

Local resection can be a management option in selected patients with middle-low rectal cancer after nCRT for LARC and without loss of oncological safety at five years.

## Introduction

In recent years, neoadjuvant chemoradiotherapy (nCRT) combined with total mesorectal resection (TME) has become the standard treatment model for locally advanced rectal cancer (LARC) [1]. LARC after nCRT may have the potential to downstage the primary tumor and even sometimes induce a pathological complete response (pCR), which occurs in 10 ~ 30% of patients [2–4], which results in lower rates of local recurrence and increased survival rates [5]. As early as 2004, Habr-Gamma reported that for patients in clinical complete response (cCR) after nCRT for LARC, the “watch-and-wait (W&W)” strategy of rectum-preservation with close follow-up and salvage surgery, if necessary, could be implemented to safeguard oncologic efficacy and save costs for health-care systems [6]. On the other hand, radical resection usually requires the creation of a temporary or permanent stoma, negatively impacting patients' quality of life (QoL). But pCR and cCR might not be equivalent, at a median 24 month follow-up, tumor regrowth was found in 24.2% of patients who underwent W&W [7]. As growing interest has developed in rectum-sparing strategies, therefore, it has been proposed that those who are cCR or near-cCR undergo local resection as a rectum-preserving strategy and decide whether to preserve the rectum according to the post-local resection ypT stage combined with other factors to avoid non-pCR patients from entering negative W&W [7].

Several studies described the effect of local resection in patients who significantly downstage to nCRT for LARC. Results shown that rectum preservation could be achieved in 64.8 to 90.0% of patients with acceptable local control [8–10]. While the findings of these studies were encouraging, the evidence of the efficacy of these approaches was still limited. Most of the studies were small, had a single-center design, differed in patients' selection and definition of clinical response and long-term oncological outcome data were scarce.

The reasons, including but not limited to ethical, patient risk, and benefit circumstances that make it challenging to develop a randomized controlled trial for local resection versus radical resection in clinical practice. There for, based on a retrospective cohort analysis of patients from two sizeable colorectal consultation centers and used a propensity-core matching (PSM) analysis to reduce the bias in patient selection between two groups, we aimed to compare long-term oncological outcomes between patients managed by local resection with those who had underwent radical resection.

## Methods

### Study design and participants

This study was a PSM and observational analysis of clinical practice across two colorectal cancer treatment centers in China [11]. We retrospectively collected information for 1911 patients with LARC after nCRT, between Jan 10, 2011, to Dec 28, 2021, from patients treated at the Fujian Medical University Union Hospital and Fujian Medical University Affiliated Zhangzhou Hospital, China.

### Inclusion and exclusion criteria

**Fig. 1:**
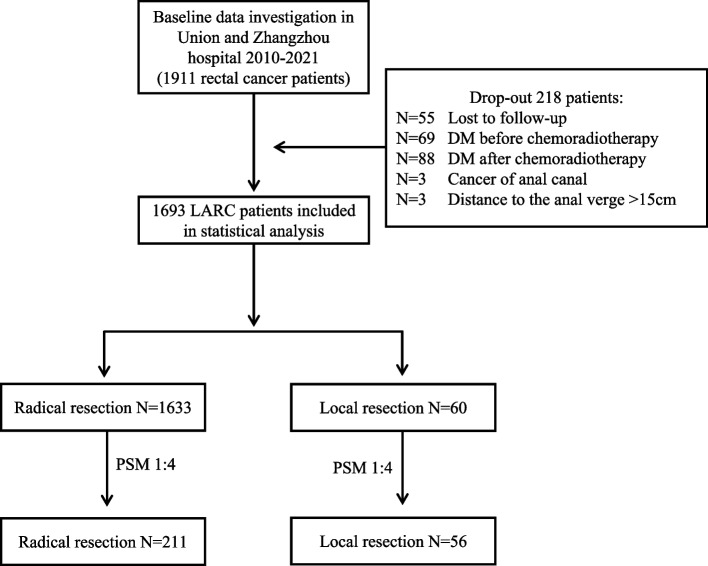
Study flow diagram. LARC locally advanced rectal cancer, DM distant metastasis

The inclusion criteria were as follows: ① pathologically confirmed rectal cancer; ② tumor response was re-evaluated after the last nCRT and the clinical stage was determined by two imaging experts as yc0 ~ III stage, which was determined according to the American Joint Committee on Cancer (AJCC) 8th edition colorectal cancer staging criteria; ③ completed nCRT combined with radical resection or local resection; ④ distance to the anal verge (DTAV) within 15 cm; ⑤ complete clinicopathological features and follow-up data, signed informed consent form of surgery [9, 10]. The exclusion criteria were as follows: ① distant organ metastasis before and after nCRT; ② death within 60 days after surgery; ③ first diagnosis combined with malignant tumors from another organ [9, 10] (Fig. 1).

### Treatment

All patients received concurrent radiotherapy and concurrent oral fluorouracil-based chemotherapy during radiotherapy. The long-course radiotherapy dose was 45.0∼50.4 Gy 25∼28 times, and the short-course radiotherapy dose was 25 Gy 5 times. The neoadjuvant chemotherapy regimen was mFolFox6 (total fluorouracil 2600 mg/m2, calcium folinate 400 mg/m2, oxaliplatin 85 mg/m2) or Xelox (capecitabine 1000 mg/m2 bid, oxaliplatin 130 mg/m2). All patients underwent magnetic resonance imaging (MRI) re-evaluated within 8–12 weeks after the end of radiotherapy. Those who were regarded by the multidisciplinary team (MDT) to have: ① re-evaluated MRI suggests a significant downstage of the tumor (ycT0 ~ 2); ② tumor invading < 30% of the intestinal circumference; ③ tumor size < 3 cm; ④ preoperative negative circumferential resection margin (CRM) and extramural venous invasion (EMVI); ⑤Tumor within 10 cm distance to anal verge; ⑥ high-middle differentiation; ⑦ no evidence of lymph node enlargement on imaging before operative, were eligible for local resection and who meet the diagnostic criteria and strongly want to preserve the anus has fully explained the disease, explained the surgical method, and local resection was performed after obtaining informed consent, whereas most of the rest patients were offered radical resection if eligible—referred to as the standard pathway. Local resection was usually performed using transanal endoscopic microsurgery (TEM) surgery, which consisted of full-thickness resection of the tumor with a margin of 2 mm or more. When histological examination revealed insufficient pathological downstaging (ypT2 ~ 3), patients were advised to underwent complete radical resection within 2 to 3 weeks after local resection unless the patient clearly knows the risks and refuses radical surgery.

### Follow-up

For patients who underwent radical resection, follow-up was following NCCN guidelines [12]. For patients managed by local resection, a more intensive follow-up protocol was used, consisting of the outpatient digital rectal examination, MRI (every 4 ~ 6 months in the first 2 years), examination under anesthesia or endoscopy, CT scan of the chest, abdomen, and pelvis, and at least two CEA measurements in the first 2 years, which was intended for early detection of possible local recurrence and/or distant metastasis (DM). For patients managed by radical resection, every three months the first three years, then every six months for the next two years, and annually after that. Physical examination, serum carcinoembryonic antigen (CEA) level, chest X-ray or computed tomography (CT), and abdominopelvic MRI or CT scans were performed at each follow-up visit. Colonoscopy was conducted annually after surgery. Positron emission tomography was performed when needed.

We calculated prognosis as the time from day 1 of concurrent surgery. local recurrence to the first recurrence at local pelvic or rectal wall, DM to the date of first distant metastasis; disease-free survival (DFS) to the first relapse at any site, death from any cause, or the date of the last follow-up visit, whichever occurred first; and overall survival (OS) to death from any cause. Followed up with patients until June 2022.

### Outcomes

The primary endpoint was to evaluate the prognostic efficacy after local excision, characterized by DFS and OS. The secondary endpoint was to evaluate the impact on local recurrence and DM.

### Statistical analysis

PSM analysis is a method used to reduce the bias in patient selection between two groups [11]. This approach attempts to construct a randomized experiment-like situation in a retrospective observational study, in which the selection bias is minimized as much as possible. We performed a PSM analysis to decrease the heterogeneity between the local resection and radical resection groups. The propensity scores of each patient were estimated using logistic regression. The following variables were included in the regression model: age, sex, ycT stage, ycN stage, ycTNM stage, radiation therapy courses, tumor regression grade (TRG), histopathology, DTAV, carcinoembryonic antigen (CEA), and carbohydrate antigen 19–9 (CA19-9). Each patient in the local resection group was matched 1:4 with a patient in the radical resection group (Caliper = 0.2). The PSM analysis was conducted using SPSS 26 (IBM SPSS Statistics, Chicago, IL, USA).

Before and after PSM, clinicopathological characteristics were compared between the two groups. The chi-Square or Fisher's exact test was used to analyze the categorical variables, whereas the rank-sum test was used for ranked data. Continuous data were compared using the student t test (data were shown as mean ± standard deviation). The survival outcomes were analyzed and compared using the Kaplan–Meier method. Multivariate analysis was performed using Cox proportional hazards regression (HR). A *p*-value < 0.05 was recognized as statistically significant.

## Results

One thousand six hundred ninety-three eligible LARC patients were enrolled to the study, 1633 of them underwent radical resection after nCRT (i.e., the standard route), and another 60 patients were performed local resection. Before PSM, there were 4 of the 60 patients with local resection who showed pathology of ypT3 after local resection, all of whom underwent additional radical resection within 2–3 weeks according to the established procedural requirements but were still included in the local resection group group in order to truly reflect the true outcome of the local resection group and to ensure the reliability of the statistical results. 4 patients died during follow-up, 2 patients developed local recurrence of the rectal wall and underwent salvage radical resection, and 4 patients developed DM.

### Patient characteristics

Baseline characteristics were shown in Table 1. Before PSM, compared with all patients who received radical resection, patients managed by local resection had tumors that were at an earlier ycT stage, ycN stage, and ycTNM stage, were less likely to had lower TRG, DTAV was lower (all *p* < 0.05). Radiotherapy doses received were clinically equivalent between treatment groups (Table 1 before PSM).

**Table 1:** Patient clinical and demographic characteristics, by treatment group (before and after PSM, *n* = 1693)

Variables	Before PSM				After PSM			
	Radical resection (*n* = 1633)	Local resection (*n* = 60)	Total	*P* value	Radical resection (*n* = 211)	Local resection (*n* = 56)	Total	*P* value
Sex				0.257				0.831
Female	802 (49.1)	25 (41.7)	827 (48.8)		90 (42.7)	23 (41.1)	113 (42.3)	
Male	831 (50.9)	35 (58.3)	866 (51.2)		121 (57.3)	33 (58.9)	154 (57.7)	
Age (years)	56.9 ± 11.6	59.4 ± 12.3	57.0 ± 11.7	0.097	59.6 ± 10.0	58.5 ± 12.0	59.4 ± 10.5	0.838
ycT stage				< 0.001				0.701
T0 ~ 1	454 (27.8)	44 (73.3)	498 (29.4)		145 (68.7)	40 (71.4)	185 (69.3)	
T2	410 (25.1)	11 (18.3)	421 (24.9)		46 (21.8)	11 (19.6)	57 (21.3)	
T3	698 (42.7)	5 (8.3)	703 (41.5)		18 (8.5)	5 (8.9)	23 (8.6)	
T4	71 (4.3)	0 (0.0)	72 (4.2)		2 (0.9)	0 (0.0)	2 (0.7)	
ycN stage				0.001				0.822
N0	1215 (74.4)	56 (93.3)	1271 (75.1)		194 (91.9)	52 (92.9)	246 (92.1)	
N +	418 (25.6)	4 (6.7)	422 (24.9)		17 (8.1)	4 (7.1)	21 (7.9)	
ycTNM stage				< 0.001				0.743
0 ~ I	760 (46.5)	53 (88.3)	813 (48.0)		181 (85.8)	49 (87.5)	230 (86.1)	
II	455 (27.9)	3 (5.0)	458 (27.1)		13 (6.2)	3 (5.4)	16 (6.0)	
III	418 (25.6)	4 (6.7)	422 (24.9)		17 (8.1)	4 (7.1)	21 (7.9)	
Radiation therapy				0.549				0.976
Long courses	1534 (93.9)	58 (96.7)	1592 (94.0)		206 (97.6)	54 (96.4)	260 (97.4)	
Short courses	99 (6.1)	2 (3.3)	101 (6.0)		5 (2.4)	2 (3.6)	7 (2.6)	
TRG				< 0.001				0.721
0	357 (21.9)	41 (68.3)	398 (23.5)		129 (61.1)	37 (66.1)	166 (62.2)	
1	542 (33.2)	13 (21.7)	555 (32.8)		59 (28.01.2)	13 (23.2)	72 (27.0)	
2	564 (34.5)	5 (8.3)	569 (33.6)		21 (10.0)	5 (8.9)	26 (9.7)	
3	170 (10.4)	1 (1.7)	171 (10.1)		2 (0.9)	1 (1.8)	3 (1.1)	
Histopathology				0.053				1.000
Adenocarcinoma	1410 (86.3)	57 (95.0)	1467 (86.7)		199 (94.3)	53 (94.6)	252 (94.4)	
Mucinous adenocarcinoma	223 (13.7)	3 (5.0)	226 (13.3)		12 (5.7)	3 (5.4)	15 (5.6)	
DTAV (cm)				< 0.001				0.727
< 5	364 (22.3)	39 (65.0)	403 (23.8)		119 (56.4)	35 (62.5)	154 (57.7)	
5∼10	1188 (72.7)	21 (35.0)	1209 (71.4)		89 (42.2)	21 (37.5)	110 (41.2)	
> 10	81 (5.0)	0 (0.0)	81 (4.8)		3 (1.4)	0 (0.0)	3 (1.1)	
CEA (ng/ml)	3.2 ± 2.3	3.5 ± 2.4	3.2 ± 2.4	0.283	2.8 ± 1.8	3.6 ± 2.0	2.9 ± 1.9	0.129
CA19-9 (U/ml)	13.7 ± 9.7	12.5 ± 10.1	13.7 ± 9.7	0.357	13.0 ± 10.1	13.0 ± 10.2	13.0 ± 10.1	0.982

Data are number (%), mean ± standard deviation, and median (p25, p75), unless otherwise specified

*PSM* Propensity-score matching, *CEA* Carcinoembryonic antigen, *CA19-9* Carbohydrate antigen 19–9, *DTAV* Distance to the anal, *TRG* Tumor regression grade

We derived paired cohorts for local resection versus radical resection (60 and 211 patients, respectively). These cohorts were well matched for key confoundersie, sex, age, ycT stage, ycN stage, ycTNM stage, TRG, histopathology, radiation therapy, CEA, CA19-9 and DTAV and were all comparable. Among locally resection patients, 87.5% (49/56) in yc0 ~ I stage, 5.4% (3/56) in yc II stage and 7.1% (4/56) in yc III stage, with DTAV less than 10 cm in all patients (Table 1 after PSM).

After PSM, Among locally resection patients, 66.1% (37/56) in ypT0, 7.1% (4/56) in ypT1, 19.6% (11/56) in ypT2 and 7.1% (4/56) in ypT3. Pathological assessment of lymph node metastasis status could not be obtained in locally resected patients and was determined to be Nx postoperatively. Patients who underwent radical resection had negative circumferential margins and complete resection of the tumor was obtained. During follow-up, there was no significant difference in the probability of local recurrence, DM, DFS, or death between the two groups (*P* > 0.05) (Table 2).

**Table 2:** Pathological and oncological outcomes after PSM in both groups (*n* = 267)

Variables	Radical resection (*n* = 211)	Local resection (*n* = 56)	Total	*P* value
ypT stage				0.502
T0	130 (61.6)	37 (66.1)	167 (62.5)	
T1	16 (7.6)	4 (7.1)	20 (7.5)	
T2	45 (21.3)	11 (19.6)	56 (21.0)	
T3	18 (8.5)	4 (7.1)	22 (8.2)	
T4	2 (0.9)	0 (0.0)	2	
ypN stage				No value
N0	194 (91.9)	—	194 (72.7)	
N +	17 (8.1)	—	17 (6.4)	
Nx	—	56 (100.0)	56 (21.0)	
Local recurrence				1.000
yes	9 (4.3)	2 (3.6)	11 (4.1)	
no	202 (95.7)	54 (96.4)	256 (95.9)	
Distant metastasis				0.521
yes	21 (10.0)	4 (7.1)	25 (9.4)	
no	190 (90.0)	52 (92.9)	242 (90.6)	
Disease-free survival				0.821
yes	184 (87.2)	50 (89.3)	234 (87.6)	
no	27 (12.8)	6 (10.7)	33 (12.4)	
Deaths				0.950
yes	18 (8.5)	4 (7.1)	22 (8.2)	
no	193 (91.5)	52 (92.9)	245 (91.8)	

### Survival outcomes

**Fig. 2:**
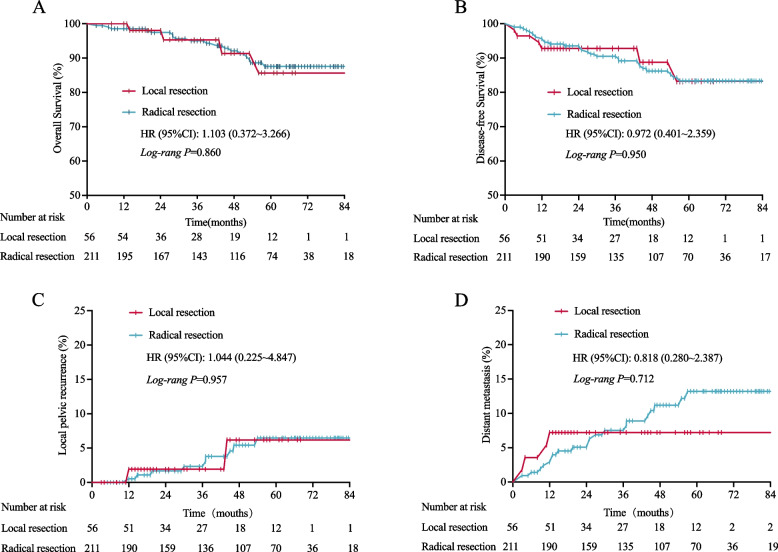
Kaplan–Meier curve of overall survival (**A**), disease-free survival (**B**), local recurrence (**C**), and distant metastasis (**D**) after propensity-score matching

The median follow-up times were 44.0 months (interquartile range = 4 ~ 107 months). In the Kaplan–Meier curves, after PSM, local resection or radical resection was not significantly associated with 1-, 3-, and 5-year cumulative incidence of OS (HR = 1.103, 95% CI: 0.372 ~ 3.266), DFS (HR = 0.972, 95% CI: 0.401 ~ 2.359), local recurrence (HR = 1.044, 95% CI: 0.225 ~ 4.847), and DM (HR = 0.818, 95% CI: 0.280 ~ 2.387) (all log-rank *P* > 0.05) (Fig. 2A ~D).

### Independent prognostic factors

We tested whether local resection was inferior to radical resection in the time-varying Cox model. Before PAM, the multivariate Cox regression analysis of OS and DFS were shown in Table 3, the sex, ycTNM stage, CA19-9, TRG, and DTAV has an independent effect on both OS and DFS. However, local resection was not an independent risk factor for OS (HR = 1.291, 95% CI: 0.466 ~ 3.547, *p* = 0.623) and DFS (HR = 1.189, 95% CI: 0.517 ~ 2.733, *p* = 0.683) noted not significantly differences.

**Table 3:** Univariate and multivariate Cox regression analyses associated of factors with OS and DFS before PSM (*n* = 1693)

Variables	OS				DFS			
	Univariate analysis HR (95% CI)	*P value*	Multivariate analysis HR (95% CI)	*P* *value*	Univariate analysis HR (95% CI)	*P value*	Multivariate analysis HR (95% CI)	*P value*
Surgery (Local vs. Radical resection)	1.291 (0.466 ~ 3.547)	0.623			1.189 (0.517 ~ 2.733)	0.683		
Sex (Male vs. Female)	0.744 (0.562 ~ 0.985)	0.039	0.745 (0.564 ~ 0.984)	0.038	0.791 (0.628 ~ 1.995)	0.045	0.792 (0.630 ~ 0.996)	0.046
Age (years)	1.013 (1.001 ~ 1.025)	0.033	1.013 (1.001 ~ 1.025)	0.027	1.006 (0.996 ~ 1.016)	0.218		
ycT stage (T4, T3, T2 vs. T0 ~ 1)	1.127 (0.859 ~ 1.481)	0.388			1.116(0.889 ~ 1.400)	0.345		
ycN stage (N + vs. N0)	0.913 (0.425 ~ 1.963)	0.815			1.048 (0.553 ~ 1.984)	0.886		
ycTNM stage (III ~ II vs. 0 ~ I)	2.111 (1.206 ~ 3.694)	0.009	2.082 (1.721 ~ 2.519)	< 0.001	1.941 (1.227 ~ 3.072)	0.005	2.060 (1.762 ~ 2.410)	< 0.001
CEA (ng/ml)	1.013 (0.963 ~ 1.065)	0.618			1.006 (0.964 ~ 1.051)	0.774		
CA19-9 (U/ml)	1.015 (1.001 ~ 1.029)	0.038	1.015 (1.002 ~ 1.029)	0.025	1.012 (1.001 ~ 1.024)	0.039	1.013 (1.002 ~ 1.025)	0.018
Radiation therapy (Long vs. Short)	0.744 (0.275 ~ 2.017)	0.561			1.951 (0.517 ~ 1.749)	0.870		
TRG (3, 2, 1 vs. 0)	1.276 (1.036 ~ 1.572)	0.022	1.373 (1.151 ~ 1.637)	< 0.001	1.211 (1.019 ~ 1.439)	0.029	1.287 (1.113 ~ 1.487)	0.001
Pathology (Adenocarcinoma vs. Mucinous adenocarcinoma)	1.103 (0.790 ~ 1.540)	0.566			1.113 (0.836 ~ 1.480)	0.464		
DTAV (> 10, 5 ~ 10 vs. < 5 cm)	0.702 (0.539 ~ 0.914)	0.009	0.700 (0.539 ~ 0.909)	0.007	0.742 (0.595 ~ 0.926)	0.008	0.741 (0.596 ~ 0.922)	0.007

*PSM* Propensity-score matching, *OS* Overall survival, *DFS* Disease-free survival, *CEA* Carcinoembryonic antigen, *CA19-9* Carbohydrate antigen 19–9, *DTAV* Distance to the anal verge, *TRG* Tumor regression grade

After PSM, only ycN stage (HR = 9.600, 95% CI: 3.938 ~ 24.405, *p* < 0.001) and DTAV (HR = 0.344, 95% CI: 0.137 ~ 0.866, *p* = 0.023) has an independent predictor of OS. Otherwise, only ycN stage (HR = 2.568, 95% CI: 1.020 ~ 6.466, *p* = 0.045) and TRG (HR = 1.729, 95% CI: 1.148 ~ 2.604, *p* = 0.009) has an independent predictor of DFS. Similarly, local excision still was not an independent risk factor for OS (HR = 0.863, 95% CI: 0.267 ~ 2.785, *P* = 0.805) and DFS (HR = 0.885, 95% CI: 0.353 ~ 2.215, *p* = 0.794) (Table 4).

**Table 4:** Univariate and multivariate Cox regression analyses of factors associated with OS and DFS after PSM (*n* = 267)

Variables	Univariate analysis, OS		Multivariate analysis, OS		Univariate analysis, DFS		Multivariate analysis, DFS	
	HR (95% CI)	*P* value	HR (95% CI)	*P* value	HR (95% CI)	*P* value	HR (95% CI)	*P* value
Surgery (Local vs. Radical resection)	0.863 (0.267 ~ 2.785)	0.805			0.885 (0.353 ~ 2.215)	0.794		
Sex (Male vs. Female)	0.573 (0.228 ~ 1.443)	0.237			0.790(0.397 ~ 1.597)	0.512		
Age (years)	1.034 (0.985 ~ 1.086)	0.174			1.032(0.994 ~ 1.071)	0.101		
ycT stage (T4, T3, T2 vs. T0 ~ 1)	1.518 (0.577 ~ 3.996)	0.398			1.016 (0.422 ~ 2.450)	0.971		
ycN stage (N + vs. N0)	7.869 (1.266 ~ 48.892)	0.027	9.600 (3.938 ~ 24.405)	< 0.001	3.417 (0.683 ~ 17.092)	0.135	2.568 (1.020 ~ 6.466)	0.045
ycTNM stage (II ~ III vs. 0 ~ I)	0.967 (0.131 ~ 7.133)	0.974			0.964 (0.176 ~ 5.275)	0.9		
CEA (ng/ml)	1.114 (0.899 ~ 1.387)	0.322			1.128 (0.940 ~ 1.353)	0.197		
CA19-9 (U/ml)	0.989 (0.946 ~ 1.033)	0.617			0.982 (0.947 ~ 1.018)	0.321		
Radiation therapy (Long vs. Short)	0.801 (0.296 ~ 2.164)	0.662			3.397 (0.730 ~ 15.806)	0.119		
TRG (3, 2, 1 vs. 0)	0.966(0.443 ~ 2.109)	0.932			1.531 (0.834 ~ 2.810)	0.169	1.729 (1.148 ~ 2.604)	0.009
Pathology (Adenocarcinoma vs. Mucinous adenocarcinoma)	1.635(0.284 ~ 9.421)	0.582			2.486 (0.625 ~ 9.885)	0.196		
DTAV (5 ~ 10 vs. < 5 cm)	0.307(0.112 ~ 0.838)	0.021	0.344(0.137 ~ 0.866)	0.023	0.513(0.250 ~ 1.051)	0.068		

*PSM* Propensity-score matching, *OS* Overall survival, *DFS* Disease-free survival, *CEA* Carcinoembryonic antigen, *CA19-9* Carbohydrate antigen 19–9, *DTAV* Distance to the anal verge, *TRG* Tumor regression grade

Owing to the potential for statistical bias due to multicollinearity, we considered that ypT and ycT staging could not be tested in the same regression model. We further included postoperative pathological outcomes in the Cox regression to verify the impact on long-term prognosis. We found that, like the previous results, the independent influences factors for OS remain to ycN stage and DTAV, for DFS remain to ycN stage and TRG in the multivariate Cox regression (all *P* < 0.05). Neither local resection nor ypT staging constituted an independent influence (Table 5).

**Table 5:** Univariate and multivariate Cox regression analyses of factors associated with OS and DFS after PSM (*n* = 267)

Variables	Univariate analysis, OS		Multivariate analysis, OS		Univariate analysis, DFS		Multivariate analysis, DFS	
	HR (95% CI)	*P* value	HR (95% CI)	*P* value	HR (95% CI)	*P* value	HR (95% CI)	*P* value
Surgery (Local vs. Radical resection)	0.954 (0.310 ~ 2.936)	0.934			0.862 (0.347 ~ 2.142)	0.750		
Sex (Male vs. Female)	0.634 (0.251 ~ 1.598)	0.334			0.817 (0.403 ~ 1.655)	0.575		
Age (years)	1.043 (0.993 ~ 1.095)	0.096			1.036 (0.998 ~ 1.076)	0.062		
ypT stage (T1 ~ 3 vs. T0)	0.763 (0.347 ~ 1.676)	0.500			0.663 (0.343 ~ 1.283)	0.222		
ycN stage (N + vs. N0)	3.835 (0.594 ~ 24.768)	0.158	9.600 (3.938 ~ 23.405)	< 0.001	2.129 (0.437 ~ 10.372)	0.350	2.568 (1.020 ~ 6.466)	0.045
ycTNM stage (II ~ III vs. 0 ~ I)	2.084 (0.302 ~ 14.366)	0.456			1.606 (0.330 ~ 7.826)	0.558		
CEA (ng/ml)	1.100 (0.887 ~ 1.364)	0.386			1.121 (0.933 ~ 1.347)	0.222		
CA19-9 (U/ml)	1.996 (0.953 ~ 1.041)	0.850			1.985 (0.950 ~ 1.022)	0.421		
Radiation therapy (Long vs. Short)	2.279 (0.239 ~ 21.692)	0.474			3.269 (0.060 ~ 15.145)	0.130		
TRG (3, 2, 1 vs. 0)	1.575 (0.583 ~ 4.251)	0.370			2.228 (1.079 ~ 4.601)	0.030	1.729 (1.148 ~ 2.604)	0.009
Pathology (Adenocarcinoma vs. Mucinous adenocarcinoma)	1.846 (0.326 ~ 10.450)	0.488			3.024 (0.772 ~ 11.848)	0.112		
DTAV (5 ~ 10 vs. < 5 cm)	0.333 (0.125 ~ 0.883)	0.027	0.344 (0.137 ~ 0.866)	0.023	0.509 (0.251 ~ 1.031)	0.061		

*PSM* Propensity-score matching, *OS* Overall survival, *DFS* Disease-free survival, *CEA* Carcinoembryonic antigen, *CA19-9* Carbohydrate antigen 19–9, *DTAV* Distance to the anal verge, *TRG* Tumor regression grade

## Discussion

In this study, we enrolled LARC patients who underwent radical resection or local resection were and PSM analysis was used to evaluate the effect of local resection on prognosis. We demonstrated that local resection was not an independent risk factor for OS and DFS after nCRT for low-middle LARC. Specifically, compared with radical resection, the oncological safety of local resection, with no significant difference in local recurrence, DM, DFS, and OS between the two groups with significant downstage of the tumor.

In the 1980s, as the report of 'Holy plane’ in rectal cancer surgery elaborated on a resection technique based on the embryologic development of the hindgut, which has tremendously improved oncologic outcomes [13]. However, these improvements come with poor QoL, especially in patients with low-middle rectal cancer [14, 15]. Organ preservation is a new concept for LARC, which HabrGama introduced in 2004.9 [6]. Similar findings with a similar therapeutic approach demonstrating reproducibility [16–18]. However, it is a lack of reliable preoperative criteria to determine whether attain cCR accurately [17]. A systemic review analysis that included 682 patients on the oncologic and survival outcomes in the W&W approach demonstrated that the 3-year cumulative risk of local regrowth was 21.6% (95% CI: 16.0 ~ 27.8); in addition, salvage surgery was performed in 88% of patients, the long-term safety of this strategy remains to be validated [19]. There for, local resection of rectal cancer can be another option for organ preservation. The advantage would be histological confirmation of whether obtain pCR. TEM surgery could be used to accurately assess pathological response in case of a complete clinical response after nCRT [8]. A multicenter, randomized trial evaluated the long-term oncological of TEM in early distal rectal cancer, the actuarial 5-year local recurrence rate was 7.7%, with 5-year DFS and OS rates of 81.6% and 82.8%, respectively, which was not difference from that in radical resection group and with improved emotional well-being (mean score at follow-up = 86.9%, 95% CI: 79.2 ~ 94.7; *P* = 0.001) [16].

In our study, there were no difference between the local resection and radical resection groups in 5-year OS, DFS, local recurrence, and DM (all log-rank *p* > 0.05). Similar results were confirmed in other studies, the GRECCAR2 was the first multicenter, randomised trial to compare local resection with radical resection in downstage low rectal cancer. Encouraging oncological results were noted at 5 years' follow-up in 5-year local recurrence (7% vs. 7%, HR = 0.71, 95% CI 0.19 ~ 2.58, *p* = 0.60), DM (18% vs. 19%, HR = 0.86, 95% CI 0.36 ~ 2.06, *p* = 0.73), OS (84% vs. 82%, HR = 0.92, 95% CI 0.38 ~ 2.22, *p* = 0.85), DFS (70% vs. 72%, HR = 0.87, 95% CI 0.44 ~ 1.72, *p* = 0.68) respectively, providing no evidence of difference in oncological outcomes between local resection and radical resection [10].

As the interest in local resection for rectal cancer increases, the accurate preoperative evaluation is extremely important, especially for lymph node metastasis status must be considered. A study utilized a nationwide cancer registry to establish incidence and predictors of nodal metastasis in early pathologic T-stage rectal cancers. The results were surprising, after receiving nCRT, LN positivity rates were 8.6% for ypT0, 12.9% for ypT1, and 21.4% for ypT2 tumors [20]. Indicated that the risk of LN metastasis increases with a higher path T stage, these findings must be carefully deliberated. Accordingly, a closer 3-year follow-up plan should be established and strictly enforced for local resection patients. Research has shown that MDT discussion contributes to improving the accuracy of MRI for the diagnosis of preoperative N stage was significantly higher than those without MDT (56.2% vs. 42.1%, *P* = 0.021), which was in favor of choosing a preferable therapy strategy [21]. Since pathologic examination of lymph node metastatic status in local resection patients will never be available, accurate preoperative evaluation as a suitable candidate for this procedure will affect the long-term prognosis of the patient, and we strongly recommend that all candidates initiate MDT discussions followed by decision making. In this study, 4 patients whose reassessment after nCRT was still highly suspicious of lymph node metastasis and rated as ycIII stage subjects underwent local resection and were required to be fully informed of the risks and offered an alternative radical resection choice before receiving this treatment.

Our study had several limitations. First, in China, there is significant heterogeneity in the occurrence of cancer and probability of death between different areas. Compared to other areas, colorectal cancer is more prevalent in the eastern area [22]. Data for this study were obtained at two large colorectal consultation centers in the eastern area of China, and characteristics may vary in other areas. Second, this study was based on a retrospective cohort analysis with some selection bias; patients with incomplete clinical data were excluded, and the probability of OS and DFS may be overestimated or underestimated. Third, there were inconsistencies in the doses of radiotherapy for patients in this study, but it has been suggested that preoperative short-course radiotherapy can also achieve excellent local control and favorable survival rates [23], and the effect of different radiotherapy doses can be effectively controlled in this study by using the baseline staging re-evaluated by MRI. Finally, post-treatment functional data were absent. In an Italian series of 46 patients managed by local resection, the data supported the hypothesis that post-treatment QoL and bowel function were better in patients managed by local resection than in those with radical resection [24]. This finding needs to be replicated in more extensive series.

Our study has several strengths. First, this was a large, reported cohort to be assessed for outcomes after local resection for LARC and the median follow-up times were 44.0 months (4–107 months). Second, this cohort represented clinical practice rather than specialist institutional practice. Third, as assessment of oncological safety requires examination of survival outcomes, comparator groups need to be matched for confounding factors, as done in our matched analysis, which was still incompletely addressed by previously randomized controls [8–10].

In conclusion, our study shown that local resection after nCRT for LARC was found to be safe. Local resection can be proposed in selected patients with middle-low rectal cancer with a significant downstage after nCRT and was not an independent risk factor for OS and DFS after nCRT for LARC. Preoperative MDT discussion and detailed evaluation are recommended for patients who are about to undergo local resection, and a close postoperative follow-up program is strictly enforced. A more extensive cohort study is warranted to validate the prognostic role of local resection for LARC after nCRT.

## Data Availability

All data obtained or analyzed during this work are included within the article.
